# Andexanet alfa for the management of severe bleeding: what should critical care physicians know about it?

**DOI:** 10.62675/2965-2774.20240178-en

**Published:** 2024-11-26

**Authors:** Felicio Savioli, Julyana Maiolino, Leonardo Rocha

**Affiliations:** 1 Hospital Alemão Oswaldo Cruz Department of Critical Care Medicine São Paulo SP Brazil Department of Critical Care Medicine, Hospital Alemão Oswaldo Cruz - São Paulo (SP), Brazil.; 2 Hospital Universitário Evangélico Department of Critical Care Medicine Curitiba PR Brazil Department of Critical Care Medicine, Hospital Universitário Evangélico - Curitiba (PR), Brazil.

Oral anticoagulants are used for the prevention and treatment of arterial and venous thromboembolism. Over the past 20 years, there has been a shift from the use of vitamin K antagonists (VKAs) to the use of direct oral anticoagulants (DOACs).^(1)^

Among DOACs, oral direct FXa inhibitors are increasingly being used for oral anticoagulation.^(2)^ The in-hospital mortality rate is nearly 30% in patients with spontaneous intracranial hemorrhage (ICH) receiving FXa inhibitors, and the risk of mortality is higher in patients on FXa inhibitors than in those not on anticoagulants.^(3)^

The reversal of DOACs is indicated in patients with severe or life-threatening bleeding. Because DOAC use is likely to increase with the introduction of generics, the availability of reversal agents for DOACs is an important medical need.^(4)^

Andexanet alfa is a specific antidote that reverses the anticoagulant effect of FXa inhibitors. Andexanet is a recombinant human FXa protein that binds with high affinity to circulating FXa inhibitors, thereby sequestering them.^(5)^

The dosing regimen for andexanet varies according to the type, dose, and time of the last dose of the FXa inhibitor. Additionally, guideline recommendations vary with regard to the dose.^(4,6-8)^ The low dose of andexanet is a 400mg IV bolus administered at 30mg/minute followed by an infusion of 4mg/minute for up to 120 minutes. Conversely, the high dose of andexanet is an 800mg IV bolus administered at 30mg/minute followed by an infusion of 8mg/minute for up to 120 minutes.^(9)^

The ANNEXA-A and ANNEXA-R trials^(10)^ were designed to assess the efficacy and safety of andexanet for the reversal of anticoagulation with apixaban or rivaroxaban in healthy volunteers. The primary endpoint was anti-FXa activity. The safety outcomes were thrombotic events and antibodies against FXa or andexanet. Among the apixaban-treated participants, anti-FXa activity was reduced by 94% among those who received an andexanet bolus compared with 21% among those who received a placebo. Among the rivaroxaban-treated participants, anti-FXa activity was reduced by 92% among those who received an andexanet bolus compared with 18% among those who received a placebo. No serious adverse or thrombotic events were reported.

ANNEXA-4 was a multicenter, prospective, single-group cohort study^(11)^ that enrolled patients with acute major bleeding within 18 hours of FXa inhibitor administration. The primary endpoints were anti-FXa activity during andexanet treatment and excellent or good hemostatic efficacy. The safety outcomes included major bleeding criteria, hemostatic efficacy, thrombotic events and death. There were 479 patients enrolled. Bleeding was predominantly intracranial in 69% of the patients and gastrointestinal in 23%. Excellent or good hemostasis occurred in 274 of the 342 evaluable patients. In the population in whom safety was evaluated, thrombotic events occurred in 10% of the patients. In 16 patients, these events occurred during treatment with prophylactic anticoagulation that began after the bleeding event. No thrombotic episodes occurred after oral anticoagulation was restarted. In that study, the authors concluded that in patients with major bleeding associated with FXa inhibitors, treatment with andexanet reduced anti-FXa activity and was associated with good or excellent hemostatic efficacy in 80% of the patients.

In the ANNEXA-I trial, patients who had taken FXa inhibitors within 15 hours before having an acute ICH were randomly assigned to receive andexanet or usual care.^(12)^ The primary endpoint was hemostatic efficacy, defined as expansion of the hematoma volume by 35% or less at 12 hours after baseline, an increase in the National Institutes of Health Stroke Scale (NIHSS) score less than 7 points at 12 hours, and no administration of rescue therapy between 3 and 12 hours. The safety endpoints were thrombotic events and death. A total of 263 patients were assigned to receive andexanet, and 267 were assigned to receive usual care. Among the patients receiving usual care, 85.5% received prothrombin complex concentrate (PCC). Hemostatic efficacy was achieved in 67.0% of the patients who received andexanet and in 53.1% of those who received usual care. The median reduction in anti-FXa activity from baseline to the 1-to-2-hour follow-up was 94.5% with andexanet and 26.9% with usual care. Thrombotic events occurred in 10.3% of the patients who received andexanet and in 5.6% of those who received usual care. There were no differences between the groups in terms of the modified Rankin scale score or death within 30 days. In that study, the authors observed that in patients with ICH who were taking FXa inhibitors, andexanet resulted in better control of hematoma expansion than usual care did but was associated with more thrombotic events.

The current European guidelines recommend PCC to reverse severe bleeding related to DOACs.^(13)^ However, PCC has not been approved by the Food and Drug Administration (FDA) for the management of FXa inhibitor-associated bleeding. Furthermore, in a 2024 retrospective cohort study, Ip et al.^(14)^ compared the outcomes in patients receiving DOACs related to ICH treated with PCC and conservative management. The authors reported that, compared with conservative management, PCC was not associated with improved neurological recovery or reduced mortality at 90 days, in-hospital mortality or hematoma expansion.

**Figure 1:**
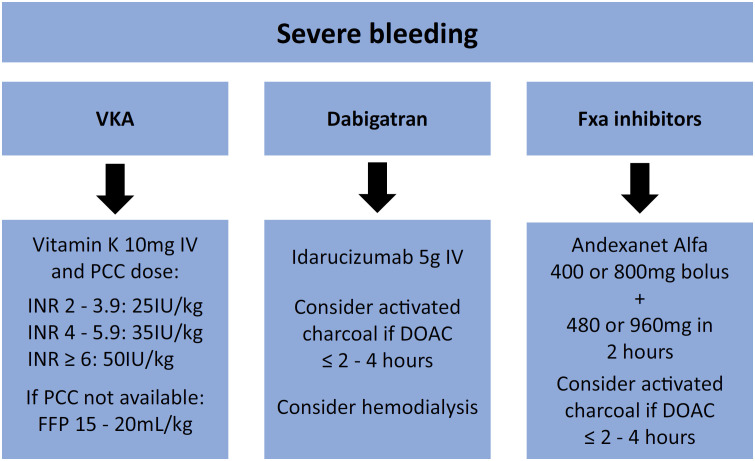
Management of severe bleeding.

We propose a flowchart (Figure 1). For severe bleeding related to VKA, the first option is the PCC, in which the dose is based on the International Normalized Ratio (INR). The second alternative is fresh frozen plasma 15 - 20mL/kg (in institutions in which PCC is not available). For severe bleeding related to dabigatran, the first option is idarucizumab (5g). Finally, hemodialysis could be an alternative. For severe bleeding related to FXa inhibitors, the first option is andexanet, a low or high dose of which can be applied on the basis of the time and dose of DOAC. Additionally, activated charcoal can be used if the final intake of any DOAC was between 2 and 4 hours prior.

VKA - vitamin K antagonist; IV - intravenous; PCC - prothrombin complex concentrate; INR - International Normalized Ratio; FFP - fresh frozen plasma; DOAC - direct oral anticoagulant.

Despite its rapid reversal of the effects of FXa inhibitors, the limiting factors for the use of andexanet are that it was made available only recently and that a single dose is costly. Further real-world studies on its use, including its cost effectiveness, are needed to enhance our knowledge of the use of this drug. Furthermore, the clinical management of severe bleeding related to DOACs is a challenge for intensive care physicians. Andexanet shows promise for reversing severe bleeding, especially ICH. However, despite its benefits in clinical trials, its use should be restricted to hospitals where hemorrhage protocols have been developed to prevent unnecessary use.
