# Supplementary material for: Andexanet alfa for the management of severe bleeding: what should critical care physicians know about it?
Source: Crit Care Sci. 2024 Nov 26;36:e20240178en. doi: 10.62675/2965-2774.20240178-en (PMC11463985; doi:10.62675/2965-2774.20240178-en)
Supplement: Translation

# Translation: Alfa-andexanete no manejo de sangramento grave: o que os intensivistas devem saber sobre isso?

**DOI:** 10.62675/2965-2774.20240178-pt

**Authors:** Felicio Savioli, Julyana Maiolino, Leonardo Rocha

**Affiliations:** 1 São Paulo SP Brasil Departamento de Cuidados Intensivos, Hospital Alemão Oswaldo Cruz - São Paulo (SP), Brasil.; 2 Curitiba PR Brasil Departamento de Cuidados Intensivos, Hospital Universitário Evangélico - Curitiba (PR), Brasil.

Os anticoagulantes orais são usados na prevenção e no tratamento de tromboembolismo arterial e venoso. Nos últimos 20 anos, houve mudança no uso de antagonistas da vitamina K (AVKs) em favor do uso de anticoagulantes orais diretos (DOACs - *direct oral anticoagulants*).^(1)^

Entre os DOACs, os inibidores diretos orais de FXa estão sendo cada vez mais usados como anticoagulantes orais.^(2)^ A taxa de mortalidade intra-hospitalar é de quase 30% em pacientes com hemorragia intracraniana (HIC) espontânea que recebem inibidores de FXa, e o risco de mortalidade é maior em pacientes que tomam inibidores de FXa do que naqueles que não tomam anticoagulantes.^(3)^

Indica-se a reversão dos DOACs em pacientes com sangramento grave ou com risco de vida. Como é provável que o uso de DOACs aumente com a introdução de genéricos, o fornecimento de agentes de reversão dos DOACs é uma necessidade médica importante.^(4)^

O alfa-andexanete é um antídoto específico que reverte o efeito anticoagulante dos inibidores de FXa. O andexanete é uma proteína FXa humana recombinante que se liga com alta afinidade aos inibidores de FXa circulantes, dessa forma sequestrando-os.^(5)^

O esquema de dosagem do andexanete varia conforme o tipo, a dose e o tempo da última dose do inibidor de FXa. Além disso, as recomendações das diretrizes variam conforme a dose.^(4,6-8)^ A dose baixa de andexanete é um bólus por via intravenosa (IV) de 400mg administrado a 30mg/minuto, seguido de uma infusão de 4mg/minuto por até 120 minutos. Por outro lado, a dose alta de andexanete é um bólus IV de 800mg administrado a 30mg/minuto, seguido de uma infusão de 8mg/minuto por até 120 minutos.^(9)^

Os estudos ANNEXA-A e ANNEXA-R^(10)^ foram desenhados para avaliar a eficácia e a segurança do andexanete na reversão da anticoagulação com apixabana ou rivaroxabana em voluntários saudáveis. O desfecho primário foi a atividade anti-FXa. Os desfechos de segurança foram eventos trombóticos e anticorpos contra FXa ou andexanete. Entre os participantes tratados com apixabana, a atividade anti-FXa foi reduzida em 94% naqueles que receberam um bólus de andexanete, em comparação com 21% naqueles que receberam placebo. Entre os participantes tratados com rivaroxabana, a atividade anti-FXa foi reduzida em 92% entre os que receberam um bólus de andexanete em comparação com 18% entre os que receberam placebo. Não foram relatados eventos adversos graves ou trombóticos.

O ANNEXA-4 trata-se de um estudo de coorte multicêntrico, prospectivo e de grupo único^(11)^ que incluiu pacientes com sangramento agudo importante dentro de 18 horas após a administração do inibidor de FXa. Os desfechos primários foram a atividade anti-FXa no tratamento com andexanete e a eficácia hemostática foi excelente ou boa. Os desfechos de segurança incluíram critérios de sangramento maior, eficácia hemostática, eventos trombóticos e morte. Foram registrados 479 pacientes. O sangramento foi predominantemente intracraniano em 69% dos pacientes e gastrintestinal em 23%. A hemostasia excelente ou boa ocorreu em 274 dos 342 pacientes avaliados. Na população em que a segurança foi avaliada, ocorreram eventos trombóticos em 10% dos pacientes. Em 16 pacientes, esses eventos ocorreram durante o tratamento com anticoagulação profilática, que começou após o evento de sangramento. Nenhum episódio trombótico ocorreu após o reinício com o anticoagulante oral. Nesse estudo, os autores concluíram que, em pacientes com sangramento maior associado a inibidores de FXa, o tratamento com andexanete reduziu a atividade anti-FXa e foi associado a uma eficácia hemostática boa ou excelente em 80% dos pacientes.

No estudo ANNEXA-I, os pacientes que tinham tomado inibidores de FXa dentro de 15 horas antes de sofrerem uma HIC aguda foram designados aleatoriamente para receber andexanete ou tratamento usual.^(12)^ O desfecho primário foi a eficácia hemostática, definida como expansão do volume do hematoma em 35% ou menos em 12 horas após o início, um aumento na pontuação da *National Institutes of Health Stroke Scale* (NIHSS) inferior a 7 pontos em 12 horas e sem administração de terapia de resgate entre 3 e 12 horas. Os desfechos de segurança foram eventos trombóticos e morte. Um total de 263 pacientes foi designado para receber andexanete e 267 foram designados para receber o tratamento usual. Entre os pacientes que receberam o tratamento usual, 85,5% receberam concentrado de complexo protrombínico (CCP). A eficácia hemostática foi alcançada em 67,0% dos pacientes que receberam andexanete e em 53,1% dos que receberam tratamento usual. A redução mediana na atividade anti-FXa basal até o seguimento de 1 a 2 horas foi de 94,5% com andexanete e 26,9% com tratamento usual. Os eventos trombóticos ocorreram em 10,3% dos pacientes que receberam andexanete e em 5,6% dos que receberam tratamento usual. Não houve diferenças entre os grupos em termos de pontuação na escala Rankin modificada ou morte em 30 dias. Nesse estudo, os autores observaram que, em pacientes com HIC em uso de inibidores de FXa, o andexanete resultou em melhor controle da expansão do hematoma do que o tratamento usual, mas foi associado a mais eventos trombóticos.

As diretrizes europeias atuais recomendam o CCP para reverter sangramentos graves relacionados aos DOACs.^(13)^ No entanto, o CCP não foi aprovado pela *Food and Drug Administration* (FDA) no manejo de sangramentos associados a inibidores de FXa. Além disso, em um estudo de coorte retrospectivo de 2024, Ip et al.^(14)^ compararam os desfechos de pacientes que receberam DOACs relacionados à HIC tratados com CCP e manejo conservador. Os autores relataram que, em comparação com o manejo conservador, o CCP não foi associado a uma melhor recuperação neurológica ou redução da mortalidade em 90 dias, mortalidade hospitalar ou expansão do hematoma.

Propomos um fluxograma (Figura 1). Na ocorrência de sangramento grave relacionado a AVK, a primeira opção é o CCP, cuja dose é calculada com base na Razão Normalizada Internacional (RNI). A segunda alternativa é de 15 a 20mL/kg de plasma fresco congelado (em instituições em que não há CCP). No caso de sangramento grave relacionado ao dabigatrana, a primeira opção é o idarucizumabe (5g). Por fim, pode-se optar pela hemodiálise. No caso de sangramento grave relacionado a inibidores de FXa, o andexanete é a primeira opção, podendo ser aplicada uma dose baixa ou alta em função do tempo e da dose de DOAC. Além disso, pode-se usar carvão ativado se a última ingestão de qualquer DOAC tiver ocorrido entre 2 e 4 horas antes.

**Figura 1:** Manejo do sangramento grave.

AVK - antagonista da vitamina K; IV - por via intravenosa; CCP - concentrado do complexo protrombínico; RNI - Razão Normalizada Internacional; PFC - plasma fresco congelado; DOAC - anticoagulante oral direto.

Apesar da rápida reversão dos efeitos dos inibidores de FXa, os fatores limitantes no uso do andexanete são sua recente introdução e o alto custo de uma única dose. Há necessidade de mais estudos reais sobre seu uso, incluindo a relação custo-benefício, para aprimorar nosso conhecimento do uso desse medicamento. Além disso, o manejo clínico do sangramento grave relacionado aos DOACs é um desafio para os intensivistas. O andexanete mostra-se promissor na reversão de sangramentos graves, especialmente a HIC. No entanto, apesar de seus benefícios em estudos clínicos, seu uso deve ser restrito a hospitais onde foram desenvolvidos protocolos de hemorragia para evitar o uso desnecessário.
